# The anatomy of the common iliac artery: a meta-analysis based on 5785 cases

**DOI:** 10.1007/s12565-024-00808-z

**Published:** 2024-11-11

**Authors:** Mateusz Koziej, Julia Toppich, Jakub Wilk, Dawid Plutecki, Patryk Ostrowski, Daniel Rams, Marta Fijałkowska, Sanjib Kumar Ghosh, Małgorzata Mazur, Renata Pacholczak-Madej, Jerzy Walocha, Michał Bonczar

**Affiliations:** 1 Department of Anatomy, Jagiellonian University Medical College Cracow, Mikołaja Kopernika 12, 33-332 Kraków, Poland; 2 Youthoria, Youth Research Organization, Kraków, Poland; 3 Jan Kochanowski University of Kielce, Kielce, Poland; 4 Department of Plastic, Reconstructive, and Aesthetic Surgery, Second Chair of Surgery Medical, University of Lodz, Łódź, Poland; 5 Department of Anatomy, All India Institute of Medical Sciences, Patna, India

**Keywords:** Common iliac artery, Abdominal aorta, Anatomy, Surgery, Spine

## Abstract

The common iliac arteries (CIA) are the main finals branches of the abdominal aorta. The aim of the present meta-analysis was to demonstrate the most up-to-date and evidence-based data regarding the general anatomy of the CIAs, including their length, take-off angles, and diameters. PubMed, Scopus, Embase, Web of Science, Cochrane Library, and Google Scholar were searched to find all studies considering the anatomy of the CIA. Eligibility assessment and data extraction stages were performed. The results of the measurements in a total of 5785 patients were evaluated and included in the statistical analysis. The prevalence of the origin variations of the CIA has been evaluated. CIA was found to most commonly originate at the level of L4 vertebrae with a pooled prevalence of 59.49% (95% CI 48.00–70.50%). The overall mean length of the CIA was 5.59 mm (SE: 0.13), and the mean diameter of the CIA was 10.52 mm (SE: 0.85). The knowledge of the anatomy and variations of the CIA is crucial in lumbar spine surgery, especially when performing the anterolateral approach to L4 and L5 vertebrae. Furthermore, the level of the aortic bifurcation has significant implications related to vascular surgery in this region. We performed the present meta-analysis to standardize the extensive information on the anatomy of the CIAs.

## Introduction

The abdominal aorta branches out into the common iliac arteries (CIAs), usually at the L4 level, which descend towards the medial aspect of the psoas major muscles and bifurcate into the external and internal iliac arteries anteriorly to the sacroiliac joint (Ellis 2010). They play a crucial role in the blood supply of the peritoneum, psoas major, and ureters (El Mamoun and Demmel 1988). Their terminal branches: internal and external iliac arteries, supply the lower extremities and pelvis with viscera (Moore et al. 2017).

The abdominal aorta and initial parts of the CIAs adjoin the superior hypogastric plexus, which supplies each CIA. The celiac, aorticorenal, superior and inferior mesenteric ganglia surround the CIAs. Moreover, coils of the small intestine are situated anteriorly to the aortic bifurcation and the CIAs. While descending, the CIAs accompany the single-named veins. Furthermore, the sympathetic trunk runs posterior to the CIA, and the ureters cross the bifurcation of the CIA. Similarly to the ureters, the superior rectal artery runs anteriorly to the left CIA (Moore et al. 2017).

The anatomy of the CIAs is subject to variabilities, mainly in their topographic and morphometric properties. Generally, the abdominal aorta bifurcates at the L4 level. However, previous studies have demonstrated that it may actually divide at other vertebral levels, namely the L3 and L5 levels and between the intervertebral discs: L3/L4 and L4/L5 (Vaccaro et al. 2012; Fataftah et al. 2020). Moreover, the right CIA is usually longer than the left because the aortic bifurcation lies to the left of the sagittal plane. The take-off angle of the CIA contributes to the differences in the vessel’s length (Nanayakkara et al. 2009). In addition, previous studies have showcased that the CIA take-off angle and length are highly variable between sexes (Shah et al. 1978; Nanayakkara et al. 2009).

With the development of lumbar spine surgery and other surgical procedures of the lower abdomen and pelvis, knowledge of the anatomy of the CIAs has become even more crucial. Performing the anterior transperitoneal and anterolateral retroperitoneal approach of the lumbar vertebrae may be burdened with potentially life-threatening complications, such as injury to the CIA (Lakchayapakorn and Siriprakarn 2008). Hence, having appropriate knowledge about the anatomy of the CIAs is of great importance (Abdul-Hameed and Ibrahim 2021).

The present study provides the most comprehensive and up-to-date synthesis of data regarding the topographical and morphometric properties of the CIA, analyzing 5785 cases. Morphometric parameters such as the length, take-off angles, and diameters of the CIAs were analyzed. Furthermore, the topography of the CIA with respect to the vertebral level was also analyzed. It is hoped that the results of the present study can be helpful for physicians, especially surgeons and interventional radiologists, when performing various surgical and endovascular procedures.

## Materials and methods

### Search strategy

PubMed, Scopus, Embase, Web of Science, Cochrane Library, and Google Scholar were searched to find all studies considering the anatomy of the CIA. The overall search process was conducted in 3 stages. In the first step, the following search terms were used in all databases: ((iliac artery) OR (common iliac artery) OR (external iliac artery) OR (internal iliac artery)) AND anatomy). Neither the date, language, type of article, nor text availability conditions were applied. (2) Furthermore, the mentioned databases were searched through once again using another set of search phrases: (a) (common iliac [Title/Abstract]) AND (anatomy [Title/Abstract]); (b) (common iliac [Title/Abstract]) AND (variation [Title/Abstract]); (c) (common iliac [Title/Abstract]) AND (morphology [Title/Abstract]); (d) (common iliac [Title/Abstract]) AND (topography [Title/Abstract]); (e) (common iliac [Title/Abstract]) AND ((branch [Title/Abstract]) OR (branches [Title/Abstract])). In addition, each phrase has been checked for dependence of results on grammatical variations of a given phrase. (3) Finally, an additional, manual search was also performed throughout all references from the initial submitted studies. Furthermore, the Preferred Reporting Items for Systematic Reviews and Meta-Analyses (PRISMA) guidelines were followed during the study. To ensure the highest quality of findings the Critical Appraisal Tool for Anatomical Meta-Analyses (CATAM) and the Anatomical Quality Assessment (AQUA) tools were used to evaluate submitted studies (Henry et al. 2017; D’Antoni et al. 2022).

### Eligibility assessment

**Fig. 1:**
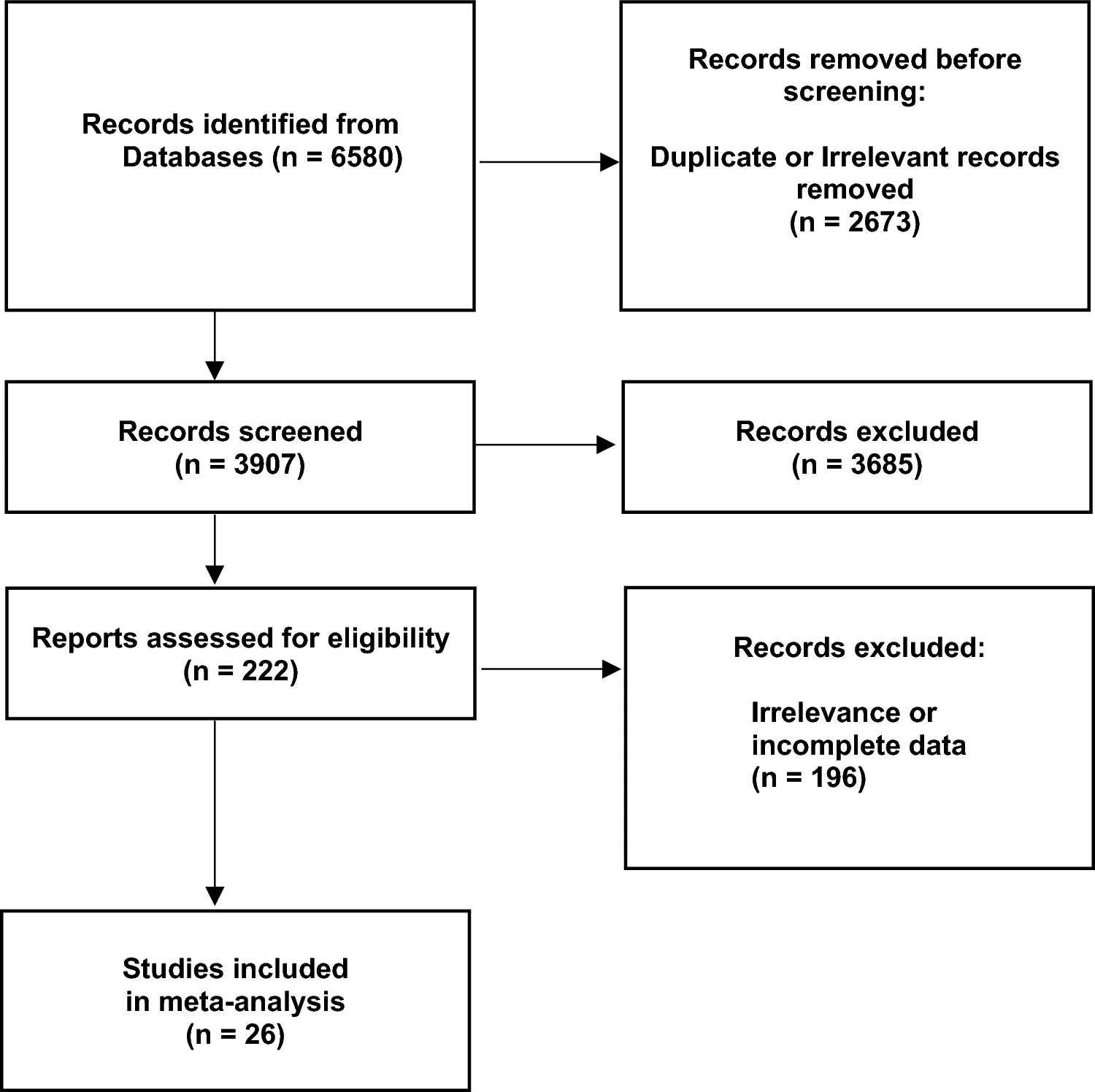
Flow diagram presenting process of collecting data included in this meta-analysis

A total of 6580 studies were initially evaluated by two independent reviewers. After comparison, 2673 records were removed as they were duplicates or irrelevant. To minimize potential bias and maintain an accurate statistical methodology, articles such as case reports, case series, conference reports, reviews, letters to the editor, and studies that provided incomplete or irrelevant data were excluded. The inclusion criteria involved original studies with extractable numerical data on anatomy, morphology, topography and variations of the CIA. Finally, 26 studies reached the criteria and were included in the present meta-analysis (Shah et al. 1978; Horejs et al. 1988; Raju et al. 1993; Kawahara et al. 1996; Ebraheim et al. 1996; Päivänsalo et al. 2000; Schep et al. 2002; Lee et al. 2004; Singh et al. 2004; Bleich et al. 2007; Khamanarong et al. 2009; Nanayakkara et al. 2009; Valecchi et al. 2010; Panagouli et al. 2011, 2021; Vaccaro et al. 2012; Joh et al. 2013; Sharma et al. 2013; Barrey et al. 2013; Deswal et al. 2014; Boonruangsri et al. 2015; Marchi et al. 2016; Wang et al. 2020; Fataftah et al. 2020; Hu et al. 2022; Minichil et al. 2022). The flow chart that describes the study inclusion process is shown in Fig. 1. The characteristics of submitted studies can be found in Table 1.

**Table 1:** Characteristics of studies included in this meta-analysis

Barrey 2013, France, Europe	
Methods	Radiological study Imaging: CT
Participants	146 patients (82 females & 64 males) 146 specimens
Outcomes	Aortic bifurcation level
Bleich 2007, USA, North America	
Methods	Cadaveric dissection
Participants	37 cadavers specimens
Outcomes	Length
Boonruangsri 2015, Thailad, Asia	
Methods	Cadaveric dissection
Participants	41 cadavers (82 specimens)
Outcomes	Length
Deswal 2014, India, Asia	
Methods	Cadaveric dissection
Participants	25 cadavers (50 specimens)
Outcomes	Length, diameter of CIA at bifurcation, angle of CIA
Ebraheim 1996, USA, North America	
Methods	Cadaveric dissection
Participants	40 cadavers (80 specimens)
Outcomes	Diameter of CIA
Fataftah 2020, Jordan, Africa	
Methods	Radiological study Imaging: CT
Participants	227 patients (106 females and 121 males)
Outcomes	Aortic bifurcation level
Horejs 1988, USA, North America	
Methods	Radiological study Imaging: CT
Participants	260 patients (130 females and 130 males) 520 specimens
Outcomes	Diameter of CIA
Hu 2022, China, Asia	
Methods	Radiological study Imaging: CT
Participants	625 patients (245 females & 380 males) 1250 specimens
Outcomes	Diameter of CIA
Joh 2013, South Korea, Asia	
Methods	Radiological study Imaging USG
Participants	1218 patients (751 females & 467 males) 2436 specimens
Outcomes	Diameter of CIA
Kawahara 1996, Japan, Asia	
Methods	Cadaveric dissection
Participants	21 cadavers specimens
Outcomes	Aortic bifurcation level
Khamanarong 2009, Thailand, Asia	
Methods	Cadaveric dissection
Participants	187 patients (55 females & 132 males) 187 specimens
Outcomes	Aortic bifurcation level
Lee 2004, South Korea, Asia	
Methods	Radiological study Imaging: MRI
Participants	210 patients (68 females & 142 males) 210 specimens
Outcomes	Aortic bifurcation level
Marchi 2016, Brazil, South America	
Methods	Radiological study Imaging: MRI
Participants	100 patients (48 females & 52 males) 200 specimens
Outcomes	Aortic bifurcation level
Minichil 2022, Ethiopia, Africa	
Methods	Radiological study Imaging: CT
Participants	136 patients (81 females & 55 males) 272 specimens
Outcomes	Diameter of CIA
Nanayakkara 2009, Sri Lanka, Asia	
Methods	Cadaveric dissection
Participants	11 cadavers (22 specimens)
Outcomes	Length, diameter of CIA at bifurcation, angle of CIA
Päivänsalo 2000, Finland, Europe	
Methods	Radiological study Imaging: USG
Participants	506 patients (255 females & 251 males) 1012 specimens
Outcomes	Diameter of CIA
Panagouli 2021, Greece, Europe	
Methods	Cadaveric dissection
Participants	76 cadavers (152 specimens)
Outcomes	Length
Panagouli 2011, Greece, Europe	
Methods	Cadaveric dissection
Participants	62 cadavers (124 specimens)
Outcomes	Length, Aortic bifurcation level
Pedersen 1993, Norway, Europe	
Methods	Radiological study Imaging: USG
Participants	160 patients (80 females & 80 males) 320 specimens
Outcomes	Diameter of CIA
Schep 2002, Netherlands, Europe	
Methods	Radiological study Imaging: MRI
Participants	16 patients (0 females & 16 males) 32 specimens
Outcomes	Diameter of CIA
Shah 1978, USA, North America	
Methods	Cadaveric dissection
Participants	26 cadavers (52 specimens)
Outcomes	Length, diameter of CIA at bifurcation, angle of CIA
Sharma 2013, India, Asia	
Methods	Cadaveric dissection
Participants	35 cadavers (70 specimens)
Outcomes	Diameter of CIA, Aortic bifurcation level
Singh 2004, Norway, Europe	
Methods	Radiological study Imaging: CT, USG
Participants	51 patients 102 specimens
Outcomes	Diameter of CIA
Vaccaro 2012, USA, North America	
Methods	Radiological study Imaging: MRI
Participants	30 patients (21 females & 9 males) 60 specimens
Outcomes	Aortic bifurcation level
Valecchi 2010, Italy, Europe	
Methods	Radiological study Imaging: USG
Participants	250 patients (125 females & 125 males) 500 specimens
Outcomes	Diameter of CIA
Wang 2020, China, Asia	
Methods	Radiological study Imaging: CT
Participants	1340 patients (626 females & 714 males) 2680 specimens
Outcomes	Diameter of CIA

### Data extraction

Data from studies that met the inclusion criteria were extracted by two independent reviewers. Qualitative data, such as data collection methodology, year of publication, and country of origin were assembled. Quantitative data about the CIA, such as prevalence of each origin in the studied group, morphometric parameters, such as length, diameter and angle of the departure were also extracted. Studies containing mean results, but without standard deviation or interquartile range or unclear or unspecified variations were excluded. Any discrepancies between the studies identified by the two reviewers were resolved by contacting the authors of the original studies wherever possible or by consensus with a third reviewer.

### Statistical analysis

To perform the meta-analyses, STATISTICA version 13.1 software (StatSoft Inc., Tulsa, OK, USA), MetaXL version 5.3 software (EpiGear International Pty Ltd., Wilston, Queensland, Australia) and Comprehensive Meta-analysis version 4.0 software (Biostat, Inc., Englewood, NJ, USA) were used. A random effects model was used in all analyses. The *χ*^2^ test and the *I*^2^ statistic were used to assess the heterogeneity among the studies (Henry et al. 2016; Higgins et al. 2019). A *p*-value and confidence intervals were used to determine statistical significance between studies. A *p*-value lower than 0.05 was considered statistically significant. In the event of overlapping confidence intervals, differences were considered statistically insignificant. *I*^2^ statistics were interpreted as follows: values of 0–40% were considered as “might not be important,” values of 30–60% were considered as “might indicate moderate heterogeneity,” values of 50–90% were considered as “may indicate substantial heterogeneity,” and values of 75–100% were considered as “may indicate substantial heterogeneity.”

## Results

**Fig. 2:**
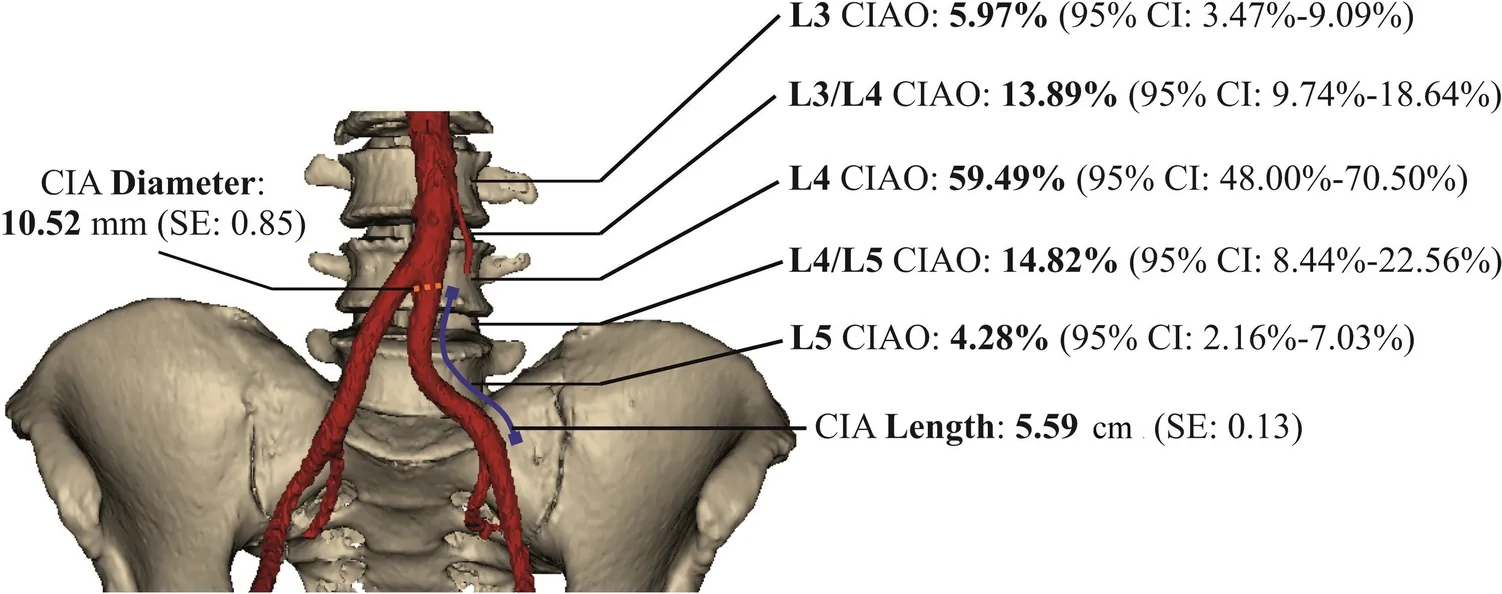
An illustration presenting the results of this meta-analysis regarding the level of origin, diameter and length of the common iliac artery (CIA). CIAO: common iliac artery origin; CI: confidence intervals; SE: standard error; CM: centimeters

The results of the measurements in 5785 patients were evaluated and included in the statistical analysis. Some of the results were illustrated and gathered in Fig. 2 to provide better readability throughout the present meta-analysis.

The prevalence of the origin variations of the CIA has been evaluated. CIA was found to most commonly originate at the level of L4 vertebrae with a pooled prevalence of 59.49% (95% CI 48.00–70.50%). The pooled prevalence of CIA originating at the level of the disc between L4 and L5 vertebrae was found to be 14.82% (95% CI 8.44–22.56%). The abovementioned results are demonstrated in Table 2.

**Table 2:** Statistical results of this meta-analysis regarding the pooled prevalence of different levels of bifurcation of abdominal aorta into common iliac arteries (CIA)

Level of aortic bifurcation	*N*	Pooled prevalence (%)	LCI (%)	HCI (%)	*Q*	I^2^
L3	740	5.97	3.47	9.09	16.51	51.55
L3/L4 (Disc)	740	13.89	9.74	18.64	19.69	59.37
L4	740	59.49	48.00	70.50	66.89	88.04
L4/L5 (Disc)	740	14.82	8.44	22.56	48.58	83.53
L5	740	4.28	2.16	7.03	16.78	52.33

LCI: lower confidence interval; HCI: higher confidence interval; Q: Cochran’s Q

The overall mean takeoff angle of the CIA from the abdominal aorta was found to be 23.44 degrees (SE: 1.74). The results were divided regarding the sex and side of the CIA. Left CIA was found to have significantly different (*p* = 0.00) takeoff angles in females and males. For all mentioned above and more detailed results, please see Table 3.

**Table 3:** Statistical results of this meta-analysis regarding the takeoff angle of the Common Iliac Artery (CIA)

Category	Mean	Standard error	Variance	Lower limit	Upper limit	*Z*-value	*p* pvalue	*p* value (between categories)
Overall	23.44	1.74	3.03	20.03	26.85	13.46	0.00	–
Left CIA	21.30	1.39	1.92	18.59	24.02	15.38	0.00	0.29
Right CIA	25.30	3.25	10.57	18.93	31.68	7.78	0.00	
Overall in females	26.13	1.85	3.43	22.51	29.76	14.11	0.00	0.27
Overall in males	21.52	2.92	8.55	15.79	27.25	7.36	0.00	
Left CIA in females	27.15	1.76	3.10	23.69	30.60	15.41	0.00	0.00
Left CIA in males	16.12	2.46	6.07	11.29	20.94	6.54	0.00	
Right CIA in females	24.68	4.30	18.48	16.26	33.11	5.74	0.00	0.80
Right CIA in males	26.28	2.81	7.91	20.76	31.79	9.34	0.00	

All measurements were established in degrees

The overall mean length of the CIA was found to be 5.59 cm (SE: 0.13). The results were divided regarding the sex and side of the CIA. The mean CIA length in females was found to be 5.49 cm (SE: 0.13), as in males was set to be 5.84 mm (SE: 0.26) No statistically significant differences were found in the subgroup analyses (*p* > 0.05). For all mentioned above and more detailed results, please see Table 4.

**Table 4:** Statistical results of this meta-analysis regarding the length of the Common Iliac Artery (CIA)

Category	Mean	Standard error	Variance	Lower limit	Upper limit	*Z*-Value	*p* value	*p* value (between categories)
Overall	5.59	0.13	0.02	5.33	5.85	41.97	0.00	–
Left CIA	5.64	0.15	0.02	5.34	5.93	37.52	0.00	0.83
Right CIA	5.58	0.21	0.05	5.16	5.99	26.19	0.00	
Overall in females	5.49	0.13	0.02	5.23	5.75	41.15	0.00	0.23
Overall in males	5.84	0.26	0.07	5.34	6.35	22.58	0.00	
Left CIA in females	5.50	0.16	0.02	5.20	5.80	35.46	0.00	0.39
Left CIA in males	5.79	0.29	0.09	5.22	6.37	19.68	0.00	
Right CIA in females	5.45	0.21	0.04	5.04	5.86	26.03	0.00	0.59
Right CIA in males	5.69	0.37	0.14	4.97	6.41	15.47	0.00	

All measurements were established in centimeters

The overall mean diameter of the CIA was found to be 10.52 mm (SE: 0.85). The results were divided regarding the sex and side of the CIA. The mean CIA diameter in females was found to be 9.47 mm (SE: 0.65), as in males was set to be 10.71 mm (SE: 0.76). No statistically significant differences were found in the subgroup analyses (*p* > 0.05). For all mentioned above and more detailed results, please see Table 5.

**Table 5:** Statistical results of this meta-analysis regarding the diameter of the Common Iliac Artery (CIA) at its origin

Category	Mean	Standard error	Variance	Lower limit	Upper limit	*Z*-Value	*p* value	*p* value (between categories)
Overall	10.52	0.85	0.72	8.85	12.18	12.39	0.00	–
Left CIA	10.16	0.91	0.83	8.37	11.95	11.14	0.00	0.78
Right CIA	10.53	0.78	0.61	9.00	12.06	13.49	0.00	
Overall in females	9.47	0.65	0.42	8.20	10.74	14.60	0.00	0.25
Overall in males	10.71	0.76	0.58	9.22	12.20	14.09	0.00	
Left CIA in females	9.34	0.75	0.56	7.87	10.81	12.46	0.00	0.28
Left CIA in males	10.79	1.00	1.01	8.82	12.76	10.74	0.00	
Right CIA in females	9.61	0.83	0.68	7.99	11.23	11.63	0.00	0.21
Right CIA in males	11.24	0.85	0.72	9.58	12.89	13.27	0.00	

All measurements were established in millimeters

**Fig. 3:**
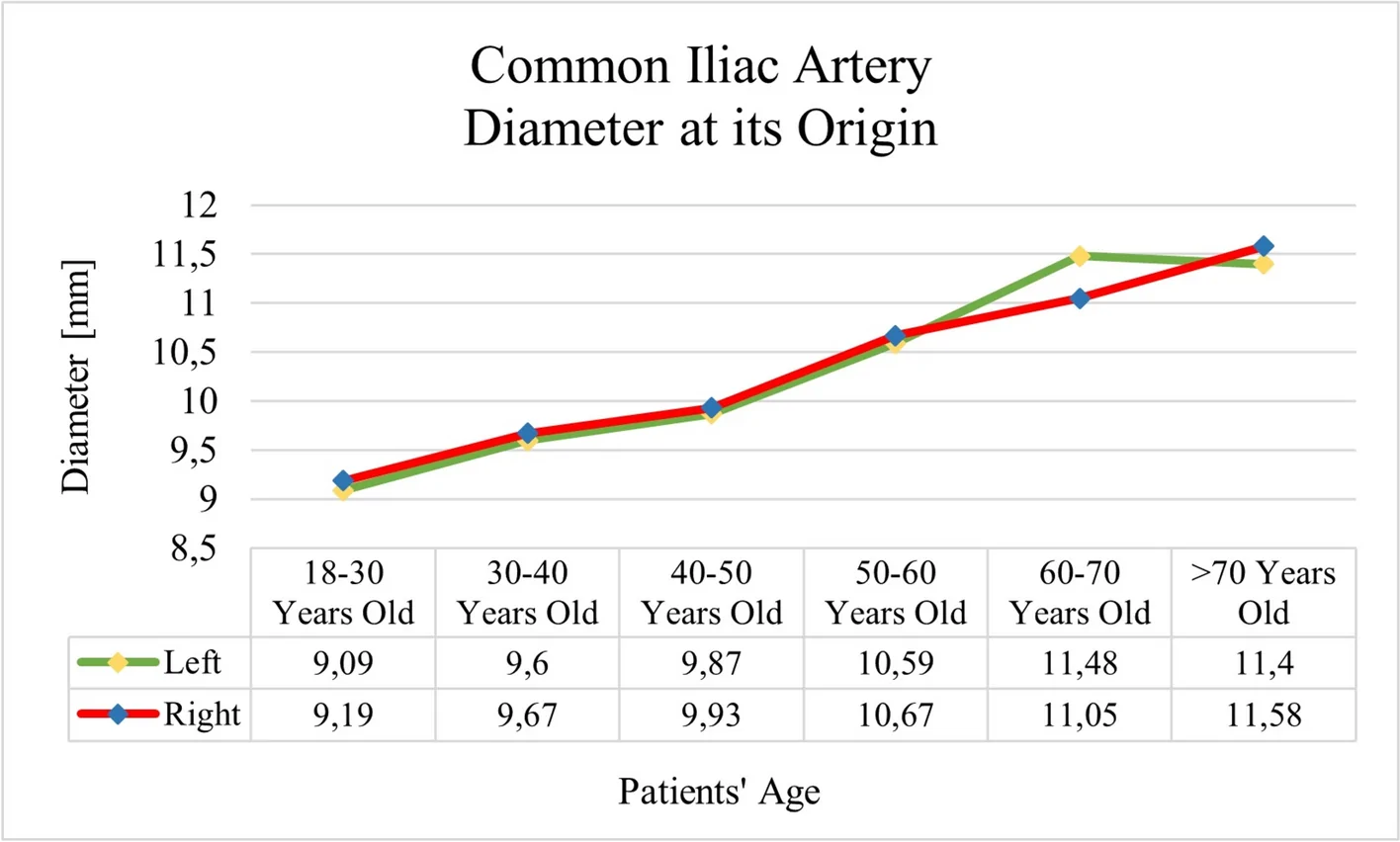
A graph illustrating the changes in the mean diameter of the Common Iliac Artery with respect to the patients’ age

Changes in CIA diameter were also evaluated with respect to the age of the patients. The group consisting of people from 60 to 70 years old was found to have the widest diameter of the left CIA (11.48 mm; SE = 1.09), whereas the group consisting of people older than 70 years old was found to have the widest diameter of the right CIA (11.58 mm; SE = 0.58). For those results please Table 6. In addition, for the graph illustrating those changes, please see Fig. 3.

**Table 6:** Statistical results of this meta-analysis regarding the diameter of the common iliac artery (CIA) at its origin, with respect to patients’ age

Category	Mean	Standard error	Variance	Lower limit	Upper limit	*Z*-Value	*p* value	*p* value (between categories)
18–30 years old								
Left	9.09	1.29	1.68	6.56	11.63	7.02	0.00	0.96
Right	9.19	1.19	1.42	6.86	11.53	7.73	0.00	
30–40 years old								
Left	9.60	0.73	0.54	8.16	11.03	13.09	0.00	0.95
Right	9.67	0.69	0.47	8.33	11.02	14.12	0.00	
40–50 years old								
Left	9.87	0.74	0.55	8.41	11.33	13.27	0.00	0.96
Right	9.93	0.71	0.51	8.54	11.33	13.93	0.00	
50–60 years old								
Left	10.59	0.72	0.52	9.18	12.00	14.74	0.00	0.94
Right	10.67	0.72	0.53	9.25	12.09	14.72	0.00	
60–70 years old								
Left	11.48	1.09	1.19	9.34	13.62	10.51	0.00	0.76
Right	11.05	0.69	0.47	9.70	12.40	16.07	0.00	
> 70 years old								
Left	11.40	0.53	0.28	10.37	12.44	21.62	0.00	0.83
Right	11.58	0.58	0.34	10.44	12.72	19.89	0.00	

All measurements were established in millimeters

## Discussion

The arterial anatomy of the posterior abdomen and pelvis is highly complex, mainly due to its variability (Kostov et al. 2021; Ongidi et al. 2021; Yevstifeieva et al. 2021; Khan et al. 2021; Zarzecki et al. 2022; Ostrowski et al. 2023; Gabryszuk et al. 2023). The CIAs are the terminal branches of the abdominal aorta. Our results demonstrate that in 59.49% of cases, the abdominal aorta bifurcates into the right and left CIAs at the L4 level to the left of the middle sagittal plane. Most studies included in this meta-analysis showed similar results. However, our meta-analysis provides a more robust pooled prevalence based on a larger cohort. Nonetheless, Kawahara et al. (1996) provided different conclusions; in nine out of 21 patients, the abdominal aorta gave rise to CIAs at the level of L4/L5 disc, while only in eight cases the CIA originated at the L4 level. Lee et al. (2004) reported that the abdominal aorta bifurcates at the L4 level in 83% of patients. Similarly, in a study conducted by Vaccaro et al. (2012), it was stated that the aortic bifurcation is located at the L4 level in 73% in the supine position, while in the prone position, it decreased to 60%. They accentuated the need to acknowledge the impact of the patient’s position on the topography of the arterial anatomy in the abdomen and pelvis. The patient’s position during lumbar spine surgery may affect the entire procedure, so being aware of this phenomenon may prevent possible arterial complications. The least common bifurcation levels were found to be L3 and L5 (Vaccaro et al. 2012; Marchi et al. 2016; Fataftah et al. 2020).

After originating from the abdominal aorta, the left and right CIAs descend towards the medial aspect of the psoas major muscles. While descending, they bifurcate into the internal and external iliac arteries. One of the most notable findings in our study is the significant variation in the take-off angles between males and females, and between the left and right CIAs. Specifically, females were found to have a significantly larger take-off angle (26.13°) compared to males (21.52°), with the right CIA consistently showing a larger angle than the left (25.30° vs. 21.30°, respectively). Interestingly, a statistically significant difference (*p* < 0.05) between the left CIA in females (27.15°) and the left CIA in males (16.12°) was found. These findings, although showcased by previous studies (Shah et al. 1978) (Deswal et al. 2014), provide more precise clinical implications due to our larger sample size. A larger take-off angle in females may make them more susceptible to arterial damage during procedures where arterial mobilization is required, such as during anterior lumbar interbody fusion (Quiñones-Hinojosa 2012). A larger take-off angle could result in greater tension or strain on the artery when it is manipulated, making it more prone to injury.

According to our research, the mean length of the CIA is 5.59 cm and the mean diameter is 10.52 mm. Interestingly, our study also demonstrated age- and sex-related differences in CIA length and diameter. In males, the CIA was generally longer (5.84 cm vs. 5.49 cm in females), with a larger diameter (10.71 mm vs. 9.47 mm in females). Moreover, our research showed that the diameter of the right CIAs is generally larger (10.53 mm) than of the left CIA (10.16 mm). However, Vallecchi et al. demonstrated (Valecchi et al. 2010) that the mean diameters of the right and left CIA are equal and amount to 9.2 mm. Chiam et al. (2013) examined the CIA diameter to determine the usefulness of transcatheter aortic valve implantation (TAVI). In the case of inoperable and high surgical-risk patients, this method emerged as a novel treatment of choice (Leon et al. 2010; Smith et al. 2011). The increasing use of endovascular procedures, such as TAVI, relies on precise knowledge of arterial dimensions, particularly in patients with complex vascular anatomy. Our findings on the morphometric characteristics of the CIA—including length and diameter, stratified by age and sex -offer updated reference data that can assist clinicians in more effectively selecting patients for procedures that involve large vascular sheaths or stent-grafts.

As the global population ages, understanding the anatomical differences between younger and older patients becomes increasingly important for surgeons and clinicians. Our meta-analysis synthesizes data on the diameter of CIAs across various age groups, revealing significant changes associated with aging. Aging is linked to degenerative disorders that can alter anatomical structures, including the CIAs. Our findings indicate a positive correlation between CIA diameter and age. For instance, the mean diameter of the left CIA in younger patients (ages 18–30) is 9.09 mm, compared to 11.4 mm in those aged 70 and older. Similarly, the right CIA shows an increase from 9.19 mm in younger patients to 11.58 mm in older individuals. While previous studies, such as those by Wang et al. (2020) have shown that arterial diameter growth peaks around ages 50 to 60, our analysis indicates a continual increase in the right CIA diameter with age. The left CIA diameter tends to peak in the 60–70 age range, aligning with Wang’s findings. These results underscore the necessity for clinicians to consider age-related anatomical changes when choosing treatment strategies, especially in populations at higher risk for vascular complications.

In recent years, lumbar spine surgery has increasingly incorporated anterior and anterolateral approaches, particularly at the L4/L5 and L5/S1 levels. These techniques place the CIAs at risk of injury, making precise knowledge of their anatomy critical to prevent severe intraoperative complications. The anterior approach, in particular, is commonly associated with a 1.9% risk of vascular injury (Brau 2004). Given the proximity of the CIAs to the lumbar vertebrae, surgeons must pay heightened attention to the arterial anatomy in this region. Barrey et al. (2013) emphasized that as the anterior approach becomes more frequently used, understanding CIA anatomy is essential for minimizing vascular complications. Similarly, Deswal et al. (2014) stressed that a thorough understanding of patient-specific anatomy is crucial for improving surgical outcomes and safety. Our meta-analysis provides a comprehensive overview of the topography and morphology of the CIA that could directly influence lumbar spine surgeries. These findings offer clinicians a standardized reference for preoperative planning, helping to reduce the risk of major hemorrhagic complications.

However, our research is not without limitations. This study employed the research conducted by other scientists. Hence, potential incorrectness may burden our studies with bias. The majority of the studies that we refer to come from Asia. We analyzed 3713 patients from Asia, 1267 from Europe, 393 from North America, 363 from Africa, and 100 from South America. Hence, this meta-analysis reflects mainly the morphology of the CIAs in Asian people rather than the global population. Furthermore, racial, target and measurement methods analyses were not presented due to insufficient amount of data and prevention of the potential bias of the results. Although not without limitations, our meta-analysis attempts to estimate CIA morphology based on the data from the literature that meet the requirements of evidence-based anatomy.

## Conclusion

The knowledge of the anatomy and variations of the CIA is crucial in lumbar spine surgery, especially when performing the anterolateral approach to L4 and L5 vertebrae (Barrey et al. 2013). Furthermore, the level of the aortic bifurcation has significant implications related to vascular surgery in this region. We performed the present meta-analysis to standardize the extensive information on the anatomy of the CIAs. Although our meta-analysis is not without limitations, we hope it will be a helpful tool during preoperative planning prior to the aforementioned procedures, and thus, contribute to a decrease in the major intra- and postoperative complications.

## Data Availability

The data that support the findings of this study are available from the corresponding author, upon reasonable request.
