# Correction: The anatomy of the common iliac artery: a meta-analysis based on 5785 cases

**DOI:** 10.1007/s12565-024-00815-0

**Published:** 2024-12-04

**Authors:** Mateusz Koziej, Julia Toppich, Jakub Wilk, Dawid Plutecki, Patryk Ostrowski, Daniel Rams, Marta Fijałkowska, Sanjib Kumar Ghosh, Małgorzata Mazur, Renata Pacholczak-Madej, Jerzy Walocha, Michał Bonczar

**Affiliations:** 1 Department of Anatomy, Jagiellonian University Medical College Cracow, Mikołaja Kopernika 12, 33-332 Kraków, Poland; 2 Youthoria, Youth Research Organization, Kraków, Poland; 3 Jan Kochanowski University of Kielce, Kielce, Poland; 4 Department of Plastic, Reconstructive, and Aesthetic Surgery, Second Chair of Surgery Medical, University of Lodz, Łódź, Poland; 5 Department of Anatomy, All India Institute of Medical Sciences, Patna, India

**Correction: Anatomical Science International (2024)** 10.1007/s12565-024-00808-z

In this article the author’s name ‘Daniel Rams’ was incorrectly written as Daniel Ramas.

The original article has been corrected.

